# Daily acute intermittent hypoxia to improve walking function in persons with subacute spinal cord injury: a randomized clinical trial study protocol

**DOI:** 10.1186/s12883-020-01851-9

**Published:** 2020-07-08

**Authors:** Avantika Naidu, Denise M. Peters, Andrew Q. Tan, Stella Barth, Andrea Crane, Angela Link, Swapna Balakrishnan, Heather B. Hayes, Chloe Slocum, Ross D. Zafonte, Randy D. Trumbower

**Affiliations:** 1 Department of Physical Medicine and Rehabilitation, Harvard Medical School, 1575 Cambridge Street, Boston, MA 02138 USA; 2 Spaulding Research Institute, Spaulding Rehabilitation Hospital, Charlestown, MA USA; 3 Department of Rehabilitation & Movement Science, University of Vermont, Burlington, VT USA; 4 Department of Rehabilitation Medicine, School of Medicine, Emory University, Atlanta, GA USA; 5 Program in Neuroscience, Graduate School of Arts and Sciences, Harvard University, Cambridge, MA USA

**Keywords:** Intermittent hypoxia, Spinal cord injury, Spinal cord trauma, Plasticity, Low oxygen, Locomotion, Speed, Endurance, Walking

## Abstract

**Background:**

Restoring community walking remains a highly valued goal for persons recovering from traumatic incomplete spinal cord injury (SCI). Recently, studies report that brief episodes of low-oxygen breathing (acute intermittent hypoxia, AIH) may serve as an effective plasticity-inducing primer that enhances the effects of walking therapy in persons with chronic (> 1 year) SCI. More persistent walking recovery may occur following repetitive (weeks) AIH treatment involving persons with more acute SCI, but this possibility remains unknown. Here we present our clinical trial protocol, designed to examine the distinct influences of repetitive AIH, with and without walking practice, on walking recovery in persons with sub-acute SCI (< 12 months) SCI. Our overarching hypothesis is that daily exposure (10 sessions, 2 weeks) to AIH will enhance walking recovery in ambulatory and non-ambulatory persons with subacute (< 12 months) SCI, presumably by harnessing endogenous mechanisms of plasticity that occur soon after injury.

**Methods:**

To test our hypothesis, we are conducting a randomized, placebo-controlled clinical trial on 85 study participants who we stratify into two groups according to walking ability; those unable to walk (non-ambulatory group) and those able to walk (ambulatory group). The non-ambulatory group receives either daily AIH (15, 90s episodes at 10.0% O_2_ with 60s intervals at 20.9% O_2_) or daily SHAM (15, 90s episodes at 20.9% O_2_ with 60s intervals at 20.9% O_2_) intervention. The ambulatory group receives either 60-min walking practice (WALK), daily AIH + WALK, or daily SHAM+WALK intervention. Our primary outcome measures assess overground walking speed (10-Meter Walk Test), endurance (6-Minute Walk Test), and balance (Timed Up & Go Test). For safety, we also measure levels of pain, spasticity, systemic hypertension, and autonomic dysreflexia. We record outcome measures at baseline, days 5 and 10, and follow-ups at 1 week, 1 month, 6 months, and 12 months post-treatment.

**Discussion:**

The goal of this clinical trial is to reveal the extent to which daily AIH, alone or in combination with task-specific walking practice, safely promotes persistent recovery of walking in persons with traumatic, subacute SCI. Outcomes from this study may provide new insight into ways to enhance walking recovery in persons with SCI.

**Trial registration:**

ClinicalTrials.gov, NCT02632422. Registered 16 December 2015,

## Background

Persons who suffer a traumatic spinal cord injury (SCI) often must confront life-long walking deficits that limit functional independence and quality of life [1, 2]. Several studies show the importance of walking in prevention of and reduction in negative secondary health conditions (e.g., hypertension, diabetes, obesity, and bone loss etc.), greater life expectancy, and improved quality of life after injury [3–6]. Thus, for persons with incomplete SCI, treatments that promote walking recovery are highly valuable and should be prioritized in combating the deleterious sequalae that accompany SCI.

Acute intermittent hypoxia (AIH) is a relatively safe and noninvasive therapy that holds tremendous promise in promoting walking recovery in persons with SCI. Initial studies found repetitive exposures to modest AIH induced endogenous mechanisms of plasticity in respiratory motor nuclei [7, 8] and profound recovery of breathing capacity in rats with incomplete SCI as early as 7 days after injury [9]. This work led to substantial progress regarding the potential of AIH, alone or when combined with task-specific training, to also improve locomotor function following SCI. Prosser-Loose et al. (2012) showed in a rat SCI model that daily (7 consecutive days) AIH coupled with ladder-walking practice resulted in near-complete and enduring improvements in ladder-walking performance (> 4 weeks). Hayes et al. (2014) translated these findings to humans and showed that persons with chronic, incomplete SCI who received daily AIH (5 days) combined with walking practice (daily AIH + WALK) produced functionally meaningful improvements in overground walking endurance not observed with those who received daily AIH alone [10]. Navarette-Opazo later confirmed these results in a separate clinical trial and showed persistent improvements in overground walking occurred when participants with incomplete SCI received more sessions (4 weeks) of AIH combined with walking practice through treadmill training [11].

We suspect that the combination of AIH and gait training leads to an additive therapeutic effect to produce greater walking recovery improvements than either alone. Prior studies show that combinatorial therapies are effective at amplifying the effects of single treatments in persons with SCI [12, 13]. Whereas combined cellular therapies are sometimes successful in rodent iSCI models, such therapies have seldom been combined with motor training in humans due to risks of systemic drug administration [14–16]. AIH is a non-invasive treatment that may serve as a ‘primer’ for SCI rehabilitation. Traditional training therapies often require more than 4 weeks for only modest long-term benefits on function. Thus, there is considerable need for more effective approaches. Repetitive AIH may fill that need and accelerate the impact of more traditional rehabilitation strategies. While past results support this exciting possibility in persons with *chronic* SCI [10, 11], we do not yet know if daily AIH alone or when combined with walking practice has a synergistic effect on improving walking function in persons at earlier stage injuries or with less initial walking ability.

There is tremendous potential for AIH to improve locomotor function via triggering or aiding rapid forms of endogenous plasticity within residual spinal circuitry after SCI [2, 17]. Prior studies have demonstrated how the greatest locomotor recovery occurred in rats with the greatest tissue sparing (> 40%) after spinal cord contusion [18, 19]. However, Basso et al. found that only a relatively small percentage of sparing (< 2%) is sufficient to trigger neural reorganization below the spinal cord injury site to achieve functionally meaningful gains [19]. Spinal plasticity peaks within the first year after injury and thus, offers a window of opportunity for early AIH treatments to help direct neural reorganization in functionally meaningful ways [20–22].

Here, we detail the study protocol of a randomized clinical trial that examines the distinct influences of repetitive exposures (daily) to AIH, with and without walking practice, on overground walking ability in persons with subacute SCI. Using an established AIH protocol known to elicit short-term improvement of walking ability in persons with chronic spinal injuries [10], we plan to examine the potential for an extended AIH protocol to elicit safe and persistent improvements in walking ability.

### Hypothesis and aims

The fundamental hypothesis guiding this study is that repetitive exposure to AIH (10 sessions in 2 weeks) will enhance walking recovery in ambulatory and non-ambulatory persons with subacute SCI, presumably by augmenting neural plasticity through a combination of spontaneous neural recovery and AIH-mediated spinal mechanisms. To test this hypothesis, we are carrying out three specific aims: 1) to quantify the impact of daily AIH (alone) on restoring walking ability in persons with subacute SCI who are initially unable to walk overground, 2) to quantify the beneficial effects of daily AIH with walking practice (daily AIH + WALK) on improving walking ability in persons with subacute SCI who are initially able to walk overground and 3) to determine if the benefits of daily AIH in persons with subacute SCI are without evidence of maladaptive changes and pathology (e.g. hypertension, autonomic dysreflexia, pain, and spasticity) [10, 23].

## Methods

### Study design and setting

We are conducting a double-blinded, placebo-controlled, counter-balanced, randomized phase II clinical trial to assess the effects of daily AIH, with or without walking practice, on walking function in persons with subacute SCI. We are conducting this study at the Spaulding Rehabilitation Hospital with institutional review board (IRB) approval from Partners Human Research Committee and IRB approval from the Shepherd Center (Atlanta, GA).

### Sample size

We plan to study the effects of daily AIH alone and with WALK in N = 85 persons with subacute SCI. The sample size is a sum of participants who enroll in Aims 1 and 2. We established a Consolidated Standard of Reporting Trials CONSORT flow diagram to summarize our trial enrollment, intervention, allocation, follow-up, and analyses [24].

For Aim 1, our sample size computation focused on interventions: daily AIH versus daily SHAM. Data from our previous randomized clinical trial that examined the effects of daily AIH (vs daily SHAM) on walking speed in persons with chronic SCI showed an effect size of 0.84 at our final follow-up. Using this value, our estimated sample size for this aim is N = 34 participants (includes 12% attrition rate), for a sensitive non-parametric comparison of means between interventions to detect a significant difference at power > 0.8 (f = 0.6, F_1,39_ = 4.6; ρ = 0.4, α = .05).

For Aim 2, our sample size computation focused on interventions: daily AIH + WALK versus daily SHAM+WALK. Data from our previous randomized clinical trial that examined the effects of daily AIH + WALK (vs daily SHAM+WALK) on walking endurance (6MWT) in persons with chronic SCI showed a significant increase of total distance walked (131 ± 100 m). Our preliminary data of the effects of daily AIH alone showed an increase of 23.9 ± 18.0 m on the 6MWT. Using these values and under the hypothesis of an additive effect of daily AIH + WALK (vs daily SHAM+WALK, WALK), we anticipate the difference in change of walking distance between daily AIH + WALK vs daily SHAM+WALK or vs WALK will be approximately 100 m. Hence, our estimated sample size for this aim is N = 51 participants (includes 12% attrition rate), for a sensitive repeated measures ANOVA comparing interventions across 3 time points, using a pooled standard deviation of 90 m, to detect a significant difference at power > 0.8 (f = 0.6, F_1,38_ = 4.4; ρ = 0.4, α = .05).

### Study recruitment and selection

The clinical trial started in January 2016, suspended in January 2017 due to relocation of the Principal Investigator’s laboratory and re-started in January 2019. We maintain an interprofessional recruitment team that resides at Spaulding Rehabilitation Hospital. The recruitment team consists of the site principal investigator, a clinical trial coordinator, research assistants, postdoctoral research fellows, as well as, onsite physicians and research physical therapists.

We also have a Medical and Data Monitor to assess participants for significant adverse events, to assess for data integrity issues during the trial, and to determine if an adverse event requires reporting to the Principal Investigator, IRB, and Department of Defense and report integrity findings and recommendations to the Principal Investigator.

Our team realizes that recruitment is a critical component to the success of this clinical trial and is of highest priority. We established an Institutional Review Board (IRB)-approved recruitment strategy to permit a broad range of methods to recruit potential participants in our trial. This includes patient registries, site-specific clinical research networks (e.g., Spinal Cord Injury Model System, SCIMS), novel internet-based advertisement (e.g., Facebook), and website inquiry forms. We post recruitment information on relevant forums such as Spaulding’s Partners Rally, CenterWatch, and ClinicalTrials.gov and paper flyers at Spaulding Rehabilitation Hospital. Our team clinicians also review, on a daily basis, SCI diagnosis (confirmed by ICD-10 codes), demographics, and co-morbidities of patients admitted to our clinical sites. To avoid bias or coercion in the recruitment process, the team follows a written script to communicate with persons who contact us with interest in the study or whom we identify from these recruitment sources.

A. Inclusion and exclusion criteria

If the person meets our initial screening requirements, the study coordinator schedules the person for an on-site assessment to determine if they meet all inclusion and exclusion criteria (Table 1). Due to the timing of this intervention study, we anticipate that eligible participants receive ongoing rehabilitation services. We do not exclude persons from this study if they are receiving gait rehabilitation provided the treatments are not during our 2-week intervention. We do exclude persons with severe sleep-disordered breathing. To evaluate this possibility, potential participants complete one night of sleep with a portable pulse oximeter [ApneaLink®, Nonin Inc. USA]. This unit records up to 10 continuous hours of heart rate (HR), respiration rate (RR), and oxygen saturation (SpO_2_) [25]. We exclude individuals with scores of > 30 apneas and hypopneas per hour indicative of OSA [23, 26] and recommend they seek further evaluation from a certified sleep specialist.

**Table 1:**
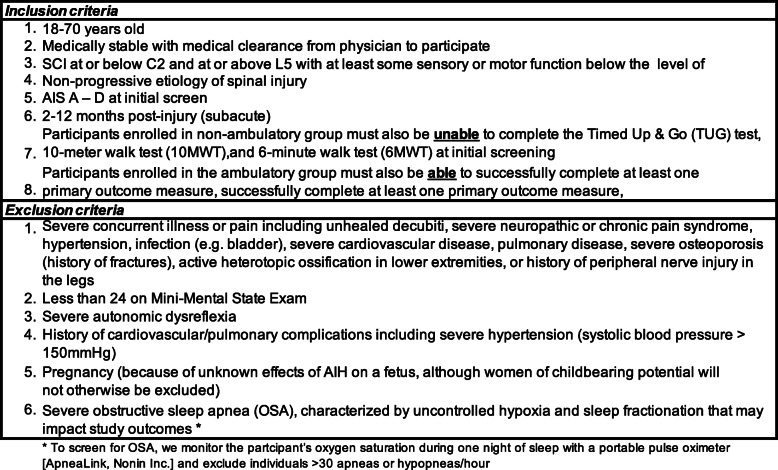
Study eligibility criteria

Since we do not yet know of any differences in responsiveness to daily AIH according to the gender or race of persons with SCI [27], we will balance our recruitment across testing groups (Aims 1 and 2) based on age, gender, and race. However, to protect against possible side effects of daily AIH, women who are pregnant or nursing a child may not take part in this study, as the effects of daily AIH on the developing fetus or infant have not been studied.

B. Informed consent process

Potential participants must read and sign the study’s consent form and Health Insurance Portability and Accountability Act (HIPAA) waiver prior to study enrollment. Our consent form incorporates the International Campaign for Cures of Spinal Cord Injury Paralysis inclusion and exclusion criteria recommendations to account for ethical considerations, safety, and potential confounds during participant recruitment [28]. Potential participants who sign the consent form undergo a medical screen by one of the study physicians and a physical screen by one of the study physical therapists. The medical screen ensures the participant meets all inclusion criteria and has no underlying medical conditions that may make them ineligible to participate. During the physical screen, the team physical therapist evaluates the individual’s functional walking ability, strength, spasticity, and pain levels using standardized clinical assessments that have high inter-rater reliability.

#### Randomization and stratification

We stratify eligible participants into either an ambulatory or a non-ambulatory group based on their initial walking function. The ambulatory group includes eligible participants who successfully complete one of the following without human assistance: TUG, 10MWT, or 6MWT without human assistance. Participants enrolled in the non-ambulatory group are unable to complete any of these assessments at initial screening. Prior to enrollment of the first study participant, a research statistician generated the balanced, randomization scheme for treatment allocation in both non-ambulatory and ambulatory groups using the R Statistical Package [29]. Using this scheme, the clinical coordinator assigns each participant to their respective treatment group using their alphanumeric identifier.

Although we inform participants about their group, we blind them to the treatment they receive (i.e. daily AIH or daily SHAM). The clinical raters, trainers, and data analysts are also unaware of the treatment that each participant receives during after the trial. We plan to rigorously quantify our blinding methods. Participants guess the breathing intervention received at the end of each treatment day and indicate guess confidence using a Likert scale [30, 31]. Using a contingency table and Fischer’s Exact Test, we will determine if the probability of correct guessing is different from chance. Using multivariate logistic regressions, we also will assess factors that may influence guessing (e.g., adverse events, perceived effects, and sensorimotor changes).

### Intervention

A. Duration

**Fig. 1:**
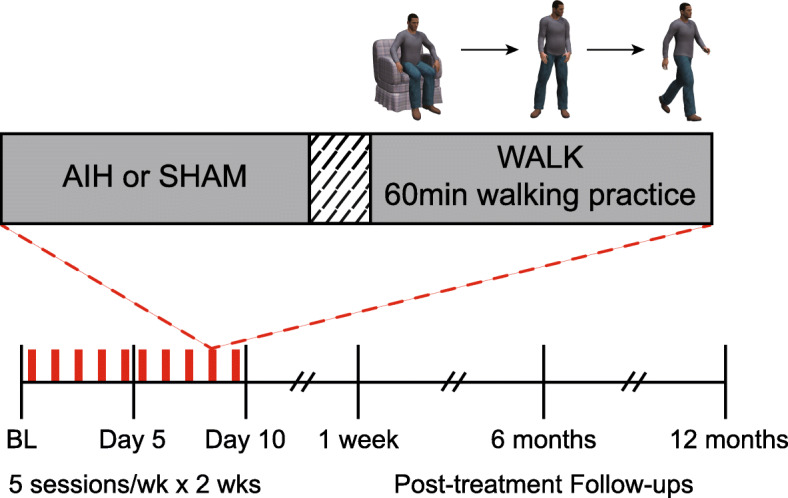
Timeline of intervention and outcome measurements

All participants enroll in 15 sessions at one of two site laboratories (1 baseline + 10 treatment days + 3 follow-up visits). Baseline (BL) evaluation occurs prior to the first day of intervention. Participants in each intervention group complete a total of ten days of intervention and 3 follow-up sessions (Fig. 1). If participants miss a treatment session, we do not conduct further assessments and exclude their data from analyses. However, if participants miss an assessment session, we record their data as “missing” at that time point.

B. Breathing treatment

Participants receive 10 treatment sessions of daily AIH or daily SHAM. A single AIH session consists of 15, 90s episodes of breathing at a fraction of inspired oxygen (FiO_2_) of 0.10 ± 0.02 with 60s intervals of 0.21 ± 0.02 FiO_2_ (room air). While a single daily SHAM session consists of 15, 90s episodes of 0.21 ± 0.02 FiO_2_ with 60s intervals of 0.21 ± 0.02 FiO_2_ (room air). We provide the treatments via a custom air delivery system; see [10, 23, 32–34] for details. In brief, the delivery system directs a known air mixture from either a pressure-swing absorption (PSA) system [HYP-123; Hypoxico Inc., USA] or a blower source to the non-rebreather facemask. For safety, oxygen concentration within the breathing circuit is continuously monitored [OM-25RME; Maxtec Inc]. Additionally, we continuously monitor blood oxygen saturation (SpO_2_) and heart rate (HR) at 1-s intervals, and blood pressure (BP) every 5th breathing interval using a MASIMO system [MASIMO rainbow SET, Irvine, CA].

C. Walking practice

Walking practice sessions immediately follow (within 10 min) the breathing intervention and last for 60 min. Walking practice involves intensive training in walking-related functional tasks using a skill-based training approach. Recent studies found skill-based walking practice is more consistent with community walking, providing meaningful gains in walking function after neurologic injury [35–37]. Task-based walking practice replicates environmental challenges encountered during real-world walking [36] and corresponds to gains in walking balance, speed, and endurance that can be measured using the timed-up and go (TUG), 10-m walk test (10MWT), and 6-min walk test (6MWT), respectively [37]. The walking practice incorporates activities to develop skills in 5 walking-related task domains: 1) walking balance (e.g., walking on different surfaces), 2) skilled walking (e.g., negotiating obstacles), 3) walking with a secondary task (e.g., walking and carrying object), 4) endurance, and 5) speed [37].

A licensed physical therapist with expertise in SCI locomotor training tailors all walking activities within each session to align with the participant’s walking ability, functional walking goals, and fatigue levels (Table 2). An overhead harness system (without provision of body weight support) is available to serve as a passive support during overground walking. Use of the harness allows participants to walk without fear of loss of balance or falling during walking practice with minimum use of hand-held walking aides. Each 60-min walking practice session is divided into two 25-min modules, involving [1] overground walking practice *with* harness support and minimum use of manual (therapist-provided) or physical (assistive device use, i.e. cane/walker/AFO) assistance, and [2] overground walking *without* harness support with use of an assistive device (if needed) by the participant and minimum use of manual assistance by the therapist. The training physical therapist ensures that participants take rest breaks as required (every 5–10 min) during training and also records SpO_2_, HR, and BP throughout the practice session.

**Table 2:**
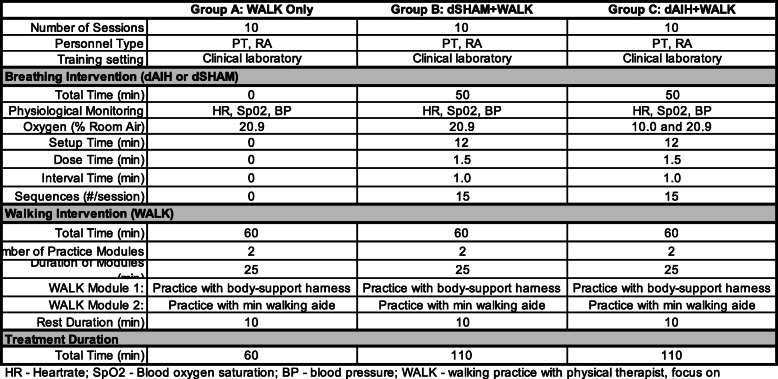
Summary of Aim 2 Experimental Groups

### Experimental protocol

D. Effects of dAIH on walking recovery in persons classified as non-ambulatory

We designed this protocol to examine if daily AIH improves walking recovery in persons with subacute SCI who are initially classified as non-ambulatory. Participants receive ten daily breathing sessions (5 days per week × 2 weeks) of either room air (daily SHAM) or daily AIH, while sitting comfortably in a chair or cushioned wheelchair. Each breathing session consists of 15 episodes of 90s hypoxia (0.10 ± 0.02 FiO_2_) for daily AIH or 90s normoxia (0.21 ± 0.02 FiO_2_; room air) for daily SHAM with 60s intervals of room air.

E. Effects of dAIH + WALK on walking recovery in persons classified as ambulatory

We designed this protocol to examine if daily AIH paired with task-specific, skilled, and intensive walking practice enhances walking recovery in persons with subacute SCI initially classified as ambulatory. Participants receive ten daily breathing sessions of either daily SHAM or daily AIH followed by daily 60-min walking practice (WALK). Our rationale is that daily AIH may be an effective pre-treatment for daily practice of walking skills, with the combined treatment being more powerful than either treatment alone, as seen in chronic SCI [10].

### Safety precautions

To ensure participant safety, we implement the following precautions:

A. *Protection against low oxygen risks during breathing sessions:* As moderate reductions in inspired oxygen may cause lightheadedness, dizziness, reduced vision, and/or euphoria, we continuously monitor SpO_2_, HR, and BP of all participants before, during, and after treatment and/or assessment sessions. If during a daily AIH treatment session a participant’s SpO_2_ levels fall below 70%, our AIH-delivery system is programmed to immediately switch from hypoxia to normal oxygen delivery (i.e. FiO2 = 0.21 O_2_) until the participant’s SpO_2_ levels re-saturate above 90%. Additionally, if participants exhibit any signs of lightheadedness, dizziness, reduced vision, and/or euphoria during a treatment session, we will immediately administer room air with our automated delivery system and the onsite study physician will conduct a clinical examination to assess if the participant should continue with treatment or terminate the session. Participants are able to discontinue treatment at any time for any reason.
B. *Protection against fall risk during walking assessments and training:* We record each participant’s fall history and document any adverse events that may occur during their training or assessment visits. To ensure safety for all walking assessments, we allow participants to use an assistive device (AD) (e.g. cane, walker, crutches etc.) depending on their level of comfort and ambulatory status, while the assessing therapist and research assistants walk beside them. We also ensure that participants use the same AD for all assessments. To ensure participant safety during all walking-training sessions, the training physical therapist constantly monitors the participant and uses a gait belt if considered clinically necessary.
C. *Protection against fatigue risk:* For all breathing sessions, we position participants in a comfortable seated or reclining position to minimize discomfort. During walking practice training, we ensure participants take seated or standing rest breaks every 5–10 min (or when requested) and between different modules. We resume training only after participants provide verbal confirmation that they are ready to continue. For all assessments, we continuously monitor participants for fatigue and balance instability, and ensure that they take frequent rest breaks (2–5 min) between different assessment tests.
D. *Protection against cardiopulmonary risk:* The assigned physical therapist (with help from a research assistant) monitors cardiopulmonary vitals (i.e. HR, BP, and SpO_2_) pre and post all assessment and treatment sessions (breathing and walking training). If a participant reports any discomfort, the on-site clinician will stop the assessment/training session for a seated/standing break (depending on participant preferences) and monitor vitals to determine if the participant’s condition is stable enough to continue after a rest period or to terminate the session altogether. The onsite clinician can also immediately withdraw participants from the study in case of serious medical issues that may arise during treatment or assessment; for example, signs of autonomic dysreflexia, rapid change in heart rate or systolic blood pressure, diaphoresis, severe headache or dizziness. All clinical and research staff members are trained to provide First Aid and CPR in case of emergency. Additionally, if a participant gets ill or injured from being in our study, a medical team (present on site for all sessions) is available to attend to the participant. Participants can return to continuing the study only after they have received medical clearance from our study physician.

### Outcome measures

We provide a summary of primary and secondary outcomes measures in Table 3. We quantify overground walking ability using three primary measures: timed up-and-go test (TUG, walking initiation and balance), ten-meter walk test (10MWT, walking speed), and six-minute walk test (6MWT, walking endurance). These tests capture functional ambulation, exhibit high reliability and construct validity, and have precedence for quantifying and distinguishing degrees of functional ambulatory recovery post SCI [38–40]. In our previous study in persons with chronic SCI, we found these tests to be more sensitive to changes in walking function and functional ambulation compared to AIS grade or categorical ambulation metrics [41]. During all walking assessments, we use a 75-ft long walkway and allow participants to use their least restrictive hand-held assistive device of choice (if needed).

**Table 3:**
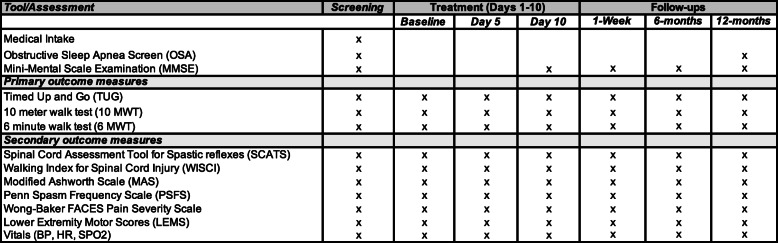
Timeline for clinical assessments and outcome measurements

We ensure a minimum of 5-min for rest breaks between tests. Participants able to ambulate attempt two trials each for the TUG and 10MWT (at their fastest, but comfortable and safe speed) with a minimum of 1-min rest between trials. The average TUG time and 10MWT speed for the two the trials will be used for analyses. Participants will attempt a single trial of the 6MWT at their fastest yet comfortable walking speed that is sustainable for six minutes, while distance covered at 2 and 6-min time points is recorded. We quantify success of completing each of the tests using a recovery score of 0–3 (0 = complete, 1 = attempted test, 2 = partially recovered but failed to complete; 3 = fully recovered). We also record secondary outcome measures of walking function that include the Walking Index for Spinal Cord Injury (WISCI) II [42] and SCI Functional Ambulation Index (SCI-FAI) [43].

To quantify maladaptive changes that may potentially occur following daily AIH exposure, we measure the magnitude and frequency of pain, spasticity, systemic hypertension, and autonomic dysreflexia during our assessments.

- Pain severity: Using the five-point Wong-Baker FACES scale of 0 (no pain) to 5 (extreme pain) [44, 45].
- Spasticity: Using the Spinal Cord Assessment Tool for Spastic Reflexes (SCATS) [46], and the cumulative sum of three SCATS subscales: clonus (0 = no spasticity; 3 = severe), flexor (0 = no spasticity; 3 = severe), and extensor (0 = no spasticity; 3 = severe); and spasms using the Penn Spasm Frequency Scale (PSFS) [47].
- Blood pressure: At all pre-post treatment/assessment and follow-up time points. For each participant we specifically record hypertension incident rate, i.e. number of hypertensive events divided by units of person-measures (the sum of the total number of BP measurements), which accounts for the total number of chances for detecting a hypertensive event and for measurements not made due to dropout or a disqualifying adverse event [48]. We will also compute relative risk for participants within each group, i.e. hypertension incidence rate in the daily AIH subgroup over that in the daily SHAM and/or WALK groups [49], respectively, with a relative risk of one indicating no association between systemic hypertension incidence with interventions.
- Autonomic dysreflexia incident rate i.e. number of autonomic dysreflexia events divided by the total person-time (number of study days completed by each participant) to account for the total number of chances for detecting autonomic dysreflexia for days not measured due to dropout or a disqualifying adverse event [48]. We also compute relative risk within each group, as autonomic dysreflexia incidence rate in the daily AIH group over the incidence rate in the daily SHAM and WALK groups, respectively [49].

### Statistical analysis

To advance our understanding and the applicability of daily AIH-induced walking recovery, we plan to test three sub-hypotheses using parametric and non-parametric statistical inferencing. Statistical significance corresponds to a p-value less than 0.05.

#### Hypothesis 1

Daily AIH improves walking recovery in persons with subacute SCI initially unable to walk as compared to daily SHAM. We plan to quantify the success of completing walking skills using our primary outcome measures (i.e., TUG, 10MWT, and 6MWT) by a recovery score of 0–3. We predict that the number of walking skills recovered will be greater for participants receiving daily AIH vs daily SHAM, indicating improved walking ability. We will use a Friedman two-way (factor 1 = intervention: daily AIH, daily SHAM; factor 2 = time: baseline, mid-test, post-test, and follow-ups) repeated measures analysis of variance (ANOVA) by ranks to compare walking recovery between and within groups. If there are significant differences, we plan post-hoc tests for pairwise comparisons [50]. We also anticipate that improvement in walking recovery will correlate with improved SCI-FAI, WISCI and LEMS scores, suggesting clinical relevance of daily AIH as an intervention across various SCI impairment levels.

#### Hypothesis 2

Daily AIH + WALK improves walking ability in ambulatory persons with subacute SCI as compared to daily SHAM+WALK and WALK. We predict a decrease in 10MWT and TUG time and an increase in 6MWT distance relative to baseline following dAIH+WALK (vs. dSHAM+WALK, WALK). We will test three related sub-hypotheses using a linear mixed model with fixed effects [51]. We will use intervention (daily AIH + WALK, daily SHAM+WALK, WALK) and time (day) as the fixed main effects, with subject as random effect, while scores for TUG (time), 10MWT (time), and 6MWT (distance) will be considered as repeated measures. Differences from baseline scores will be compared between and within interventions at mid-test, post-test, and follow-ups. If ANOVAs reveal significant differences, we will use the Tukey-Kramer post-hoc test to identify pairwise differences. If baseline measures are significantly different between intervention groups, we plan to use an analysis of co-variance (ANCOVA) to analyze these data.

#### Hypothesis 3

Daily AIH ± WALK does not induce maladaptive changes (spasticity, pain, systemic hypertension, autonomic dysreflexia) in persons with subacute SCI. First, we predict no difference in SCATS following daily AIH ± WALK as compared to daily SHAM±WALK or WALK. We also predict no difference in the changes in FACES scores between interventions. We anticipate daily AIH alone or combined with walking practice will not elicit greater incidence of systemic hypertension in persons with subacute SCI. To test this hypothesis, we will compare the incidence rates of hypertension between interventions (daily AIH, daily AIH + WALK, daily SHAM+WALK, WALK) using Relative Risk [49, 52]. A Relative Risk of one will indicate no association between systemic hypertension and interventions. Using the 95% confidence interval of the Relative Risk [49], we will determine if there is a statistically significant association between interventions. We predict the relative risk of hypertension comparing daily AIH vs. daily SHAM, daily AIH + WALK vs. daily SHAM+WALK, and daily AIH + WALK vs. WALK are not different. We also predict the incidences of autonomic dysreflexia comparing daily AIH vs. daily SHAM, daily AIH + WALK vs. daily SHAM+WALK, and daily AIH + WALK vs. WALK are not different.

### Data monitoring and management

Our data safety monitoring board (DSMB) is responsible for data monitoring, interim analyses, and auditing. We routinely update the DSMB with study progress and safety and will collect and manage all study data using the REDCap (Research Electronic Data Capture) electronic data capture tools hosted at Partners Healthcare Inc. [53, 54]. REDCap is a secure, web-based software platform designed to support data capture. This database meets all current standards for clinical trial configuration and utilization for data logging, auditing, and recovery purposes. To ensure participant confidentiality and blinding of ratings, data are de-identified using alphanumeric codes.

To minimize biases and errors in data collection, a designated lead physical therapist, blinded to study interventions, ensures consistent scoring among therapists by regularly checking all assessment logs for adherence to standard clinical procedures. Blinded research staff members (including postdoctoral fellows, a lab engineer, and research assistants) assist clinicians with training setup and clinical data collections. The study PI oversees all study procedures and ensures correct collection and documentation of data.

### Adverse event reporting

The research team (PI, clinicians, and research assistants) reports adverse events related to each participant from the time of enrollment to the last follow-up assessment visit, which include: 1) unintentional loss of balance (i.e. fall to the ground), 2) change in systolic pressure to > 140 mmHg and/or diastolic pressure exceeding > 90 mmHg [55, 56], 3) autonomic dysreflexia with systolic blood pressure > 150 mmHg or > 20 mmHg from baseline with complaints of headache, diaphoresis, and/or blurred vision, 4) musculoskeletal injury during/after walking training (i.e. sprain, fracture, etc.), 5) symptoms such as pain, soreness, numbness, or signs of injury (inflammation, blisters, etc.) during or immediately following training or on returning home, 6) hospitalization for any cause, and 7) death due to any cause.

### Study compensation

Participants receive $25 per visit. Participants who live more than 60 miles from the INSPIRE laboratory are eligible for lodging and travel reimbursement.

Study Trial Registration.

We registered the trial on ClinicalTrials.gov (Registration #: NCT02632422) prior to enrollment of study participants.

## Discussion

The goal of this study is to examine the enduring effects of daily AIH, alone or in combination with task-specific walking practice, on walking recovery in persons with traumatic, subacute SCI. Prior studies have shown that daily AIH is a potent primer of walking training, and can improve walking ability, when used alone or as a combinatorial approach, compared to training alone [10, 57]. Indeed, restoring community walking is a top priority for persons living with SCI, as improvements in walking function enables them to participate more independently in a broad range of daily living activities and combating the deleterious effects of secondary health conditions. Traditional gait training approaches offer only modest recovery of walking function in persons with SCI [58]. Thus, identification of early subacute treatments that may facilitate neural plasticity within spared spinal pathways in a safe and efficacious manner, regardless of ambulatory status, is critical [59, 60]. Follow-ups with participants up to a year after treatment ends will allow us to analyze an entire year’s worth of recovery in the subacute stage. We anticipate outcomes from this study will provide new insights into the potential clinical utility of AIH-based translational approaches to enhance functional independence in persons with SCI.

## Supplementary information

**Additional file 1.** CONSORT Diagram.

## Data Availability

Final trial data are accessible to the study investigators. However, individual data requests should be made to the principal investigator. The research team plans to disseminate results in the form of published manuscripts and presentations. We plan also to make information available via the Spinal Cord Injury Common Data Elements (CDE) standards developed through the collaboration of the International Spinal Cord Society, the American Spinal Injury Association, and the National Institutes of Health National Institute of Neurological Disorders. Per request of the funding agencies Program Office, we will submit data for USAMRMC archiving accordant with privacy policies of the USAMRMC and Institutional Review Boards at the collaborating sites: Spaulding Rehabilitation Hospital and Shepherd Center.

## Abbreviations

AIH: Acute intermittent hypoxia
BDNF: Brain-derived neurotrophic factor
FiO_2_: Fraction of inspired oxygen
O_2_: Oxygen
PSA: Pressure-swing absorption
SCI: Spinal cord injury
SE: Standard error
SHAM: Placebo treatment; room air treatment
SpO_2_: Oxygen saturation
