# Supplementary material for: Daily acute intermittent hypoxia to improve walking function in persons with subacute spinal cord injury: a randomized clinical trial study protocol
Source: BMC Neurol. 2020 Jul 8;20:273. doi: 10.1186/s12883-020-01851-9 (PMC7341658; doi:10.1186/s12883-020-01851-9)
Supplement: Supplementary file 1 — Additional file 1. CONSORT Diagram. [file 12883_2020_1851_MOESM1_ESM.pdf]

CONSORT Diagram

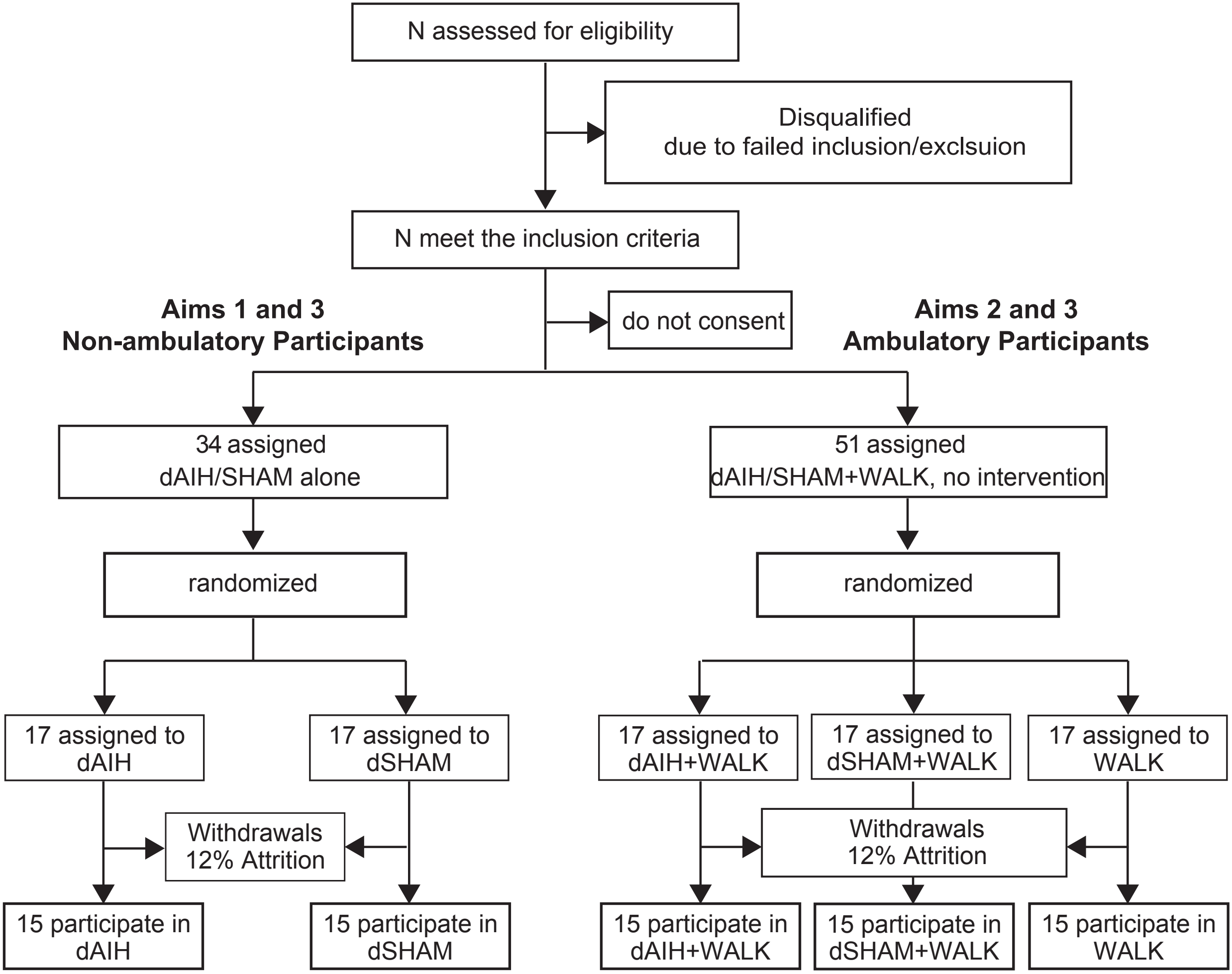

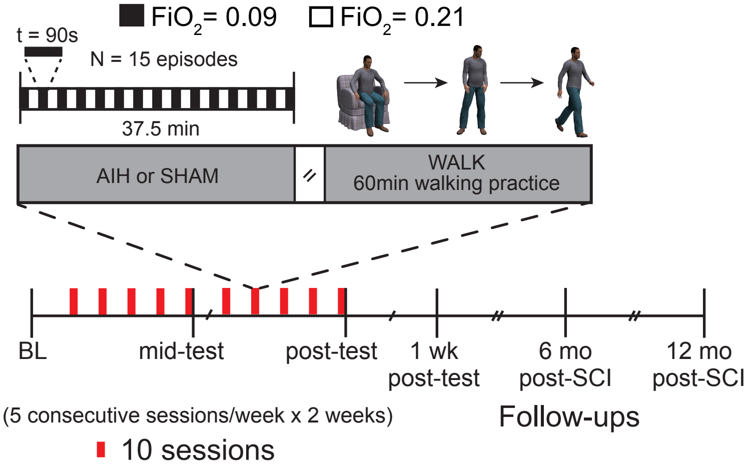

FiO2= 0.09
FiO2= 0.21
t = 90s
N = 15 episodes
37.5 min
AIH or SHAM
WALK
60min walking practice
BL
mid-test
post-test
1 wk
post-test
6 mo
post-SCI
12 mo
post-SCI
(5 consecutive sessions/week x 2 weeks)
Follow-ups
10 sessions
